# *In vitro* induction of trained immunity in adherent human monocytes

**DOI:** 10.1016/j.xpro.2021.100365

**Published:** 2021-02-24

**Authors:** Jorge Domínguez-Andrés, Rob J.W. Arts, Siroon Bekkering, Harsh Bahrar, Bastiaan A. Blok, L. Charlotte J. de Bree, Mariolina Bruno, Özlem Bulut, Priya A. Debisarun, Helga Dijkstra, Jéssica Cristina dos Santos, Anaísa V. Ferreira, Daniela Flores-Gomez, Laszlo A. Groh, Inge Grondman, Leonie Helder, Cor Jacobs, Liesbeth Jacobs, Trees Jansen, Gizem Kilic, Viola Klück, Valerie A.C.M. Koeken, Heidi Lemmers, Simone J.C.F.M. Moorlag, Vera P. Mourits, Jelmer H. van Puffelen, Katrin Rabold, Rutger J. Röring, Diletta Rosati, Helin Tercan, Julia van Tuijl, Jessica Quintin, Reinout van Crevel, Niels P. Riksen, Leo A.B. Joosten, Mihai G. Netea

**Affiliations:** 1 Department of Internal Medicine, Radboud University Nijmegen Medical Centre, Geert Grooteplein 8, 6500HB Nijmegen, the Netherlands; 2 Radboud Institute for Molecular Life Sciences (RIMLS), Radboud University Medical Center, 6525 GA Nijmegen, the Netherlands; 3 Immunology of Fungal Infections, Department of Mycology, Institut Pasteur, 75015 Paris, France; 4 Department for Genomics & Immunoregulation, Life and Medical Sciences Institute (LIMES), University of Bonn, 53115 Bonn, Germany; 5 Department of Computational Biology for Individualised Infection Medicine, Centre for Individualised Infection Medicine (CiiM) and TWINCORE, The Helmholtz Centre for Infection Research (HZI) and The Hannover Medical School (MHH), Hannover, Germany

**Keywords:** Cell biology, Cell isolation, Cell-based assays, Immunology

## Abstract

A growing number of studies show that innate immune cells can undergo functional reprogramming, facilitating a faster and enhanced response to heterologous secondary stimuli. This concept has been termed “trained immunity.” We outline here a protocol to recapitulate this *in vitro* using adherent monocytes from consecutive isolation of peripheral blood mononuclear cells. The induction of trained immunity and the associated functional reprogramming of monocytes is described in detail using β-glucan (from *Candida albicans*) and Bacillus Calmette-Guérin as examples.

For complete details on the use and execution of this protocol, please refer to Repnik et al. (2003) and Bekkering et al. (2016).

## Highlights

• Isolation of PBMCs and monocytes using discontinuous density gradients
• *In vitro* induction of trained immunity in adherent monocytes
• Induction of trained immunity is assessed by cytokine production levels
• Generally applicable to test multiple stimuli and pharmacological compounds

## Graphical abstract

**Figure:**
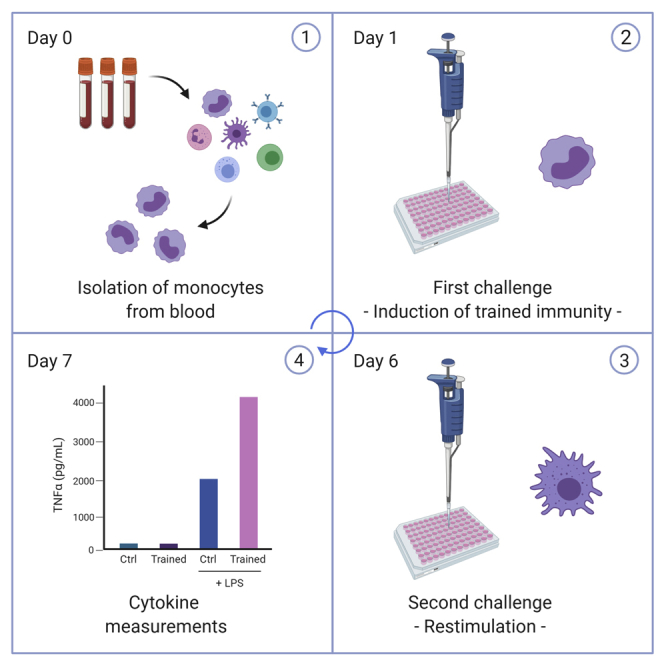

## Before you begin

### Isolation of PBMCs from a buffy coat

**Timing (day 0): 2 h approximately**

**Figure 1:**
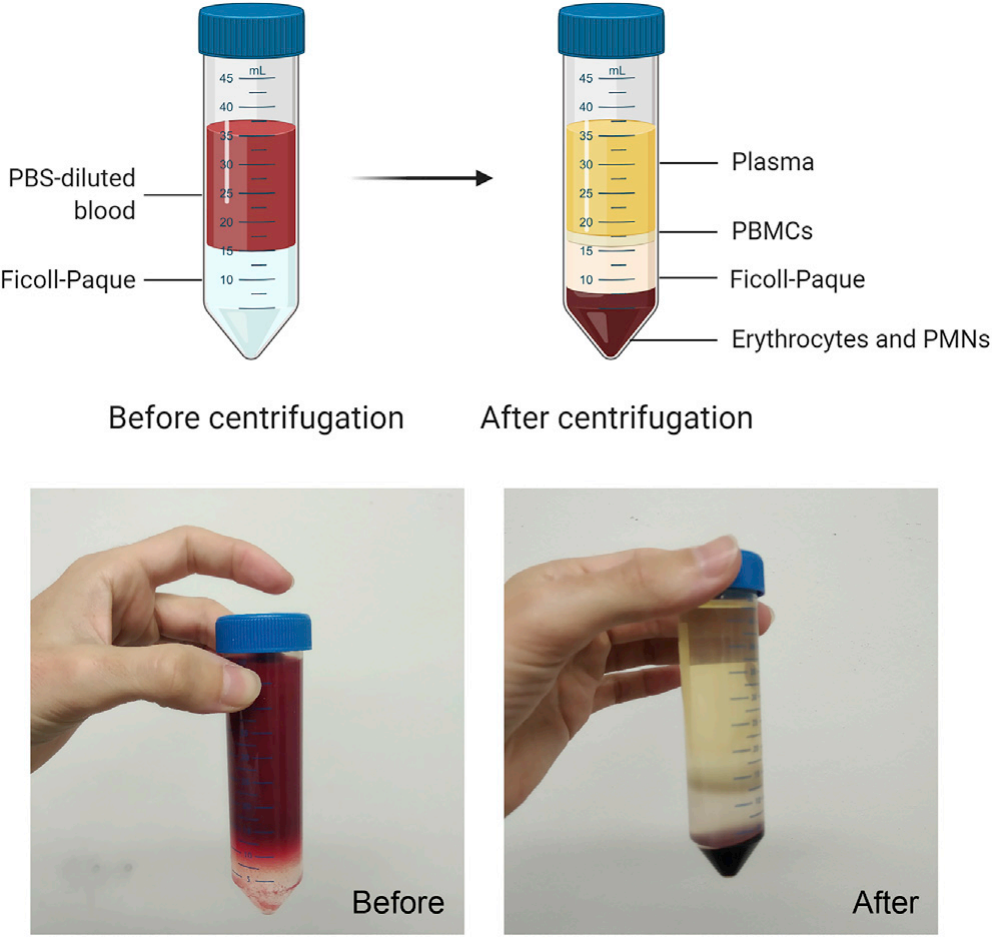
Aspect of the different layers before and after density gradient centrifugation with Ficoll-Paque

By using buffy coats it is possible to obtain large numbers of cells from the same donor. In a buffy coat the white blood cells of 500 mL of whole blood are concentrated into 50 mL. Different subsets of blood cells have different densities. This property is used to separate the fraction containing peripheral blood mononuclear cells (PBMC) from the rest of the blood. When Ficoll-Paque is pipetted underneath the whole blood, the centrifugation process allows cells with a high density to migrate through the density gradient while cells with a lower density, such as PBMCs, remain on top of the gradient and can be easily collected (Figure 1). The protocols of separation of PBMCs and monocytes from whole blood using density gradients are further described by Repnik et al. (Repnik et al., 2003).

1. Density gradient centrifugation
  a. Transfer the blood from each buffy coat into 8 conical tubes of 50 mL.
  b. Adjust the volume of each tube to 35 mL by adding PBS.
  c. Add 14 mL Ficoll-Paque underneath the PBS-blood layer using a 10 mL pipette (Methods video S1). The end volume should be 49 mL.
  d. Spin down 50 mL tubes: 615 × *g* for 30 min at 20°C acceleration 1, no break.
  e. Take the PBMC layer using a sterile Pasteur pipette. Put the PBMCs collected from each donor into 4 × 50 mL conical tubes.
  f. Add PBS to a final volume of 50 mL.

***Note:*** Do not take the cells attached to the wall (troubleshooting 1).

2. Washing steps (work on ice)
  ***Note:*** constantly check the pellet for clumps. Resuspend them using a pipette (see troubleshooting 2).
  a. Spin down tubes 550 × *g* for 15 min at 4°C
  b. Remove the supernatant and pool pellets into 2 × 50 mL conical tube per donor.
  c. Wash with cold PBS; end volume 50 mL.
  d. Spin down tubes 550 × *g* for 10 min at 4°C.
  e. Remove the supernatant and pool pellets into 1 × 50 mL conical tube per donor. Repeat steps c and d.
  f. Resuspend the cells in 30 mL of RPMI 1640 without serum using a Pasteur pipette and count the cells with a hemocytometer, or an automated cell counter system such as a CASY cell counter or a Sysmex hematology instrument.

**CRITICAL:** Slowly lay the Ficoll-Paque layer underneath the PBS-diluted blood with a 10 mL pipette using gravity (Methods video S1). Disable the break for density gradient centrifugation since breaking can disrupt the PBMC layer.

***Note:*** Alternatively, PBS-diluted blood can be slowly laid on top of the Ficoll-Paque as well using a 25 mL stripette.

Methods video S1. How to lay the Ficoll-Paque layer underneath the PBS-diluted blood, related to isolation of PBMCs from a buffy coat

### Monocyte isolation with Percoll

**Timing (day 0): 2 h approximately**

After Ficoll-Paque isolation of monocytes and lymphocytes, it is possible to separate monocytes from lymphocytes by using a hyper-osmotic density gradient medium; Percoll. Separation of the two cell types is feasible since lymphocytes are more sensitive to an increase in osmolarity than monocytes.

For this step, prepare hyper-osmotic Percoll solution in advance: For 100 mL of solution, mix 48.5 mL of Percoll, 41.5 mL of sterile water and 10.0 mL of 0.22 μm filter-sterilized 1.6 M NaCl and shake vigorously.

**Figure 2:**
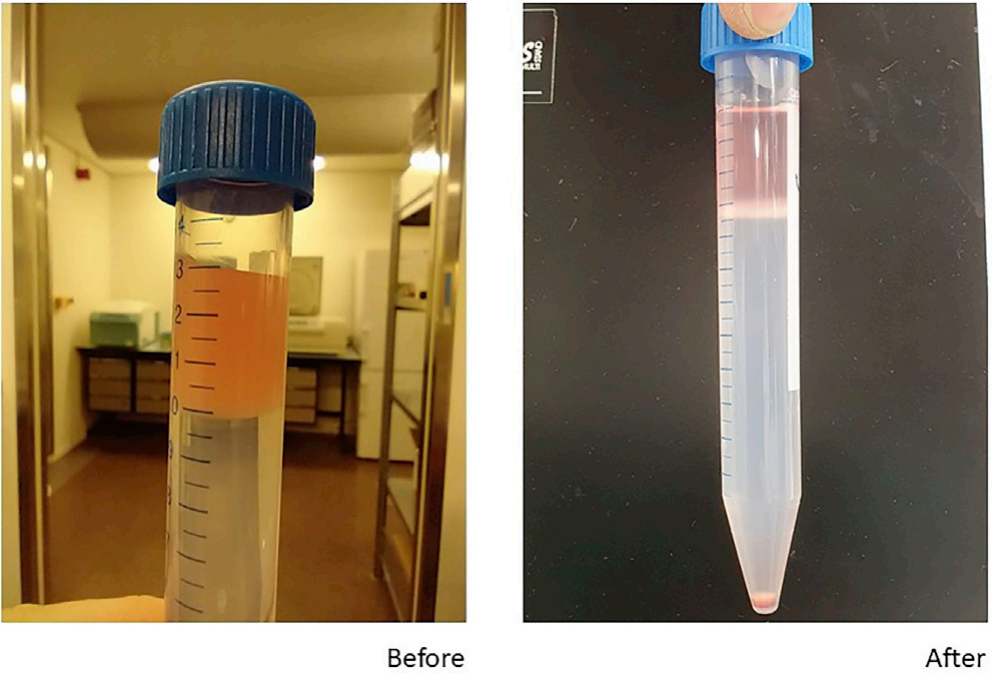
Aspect of the different layers before and after density gradient centrifugation with Percoll
  b. Do not collect the last 4 mL.
  c. Combine the cells from up to three tubes. Add cold PBS to a final volume of 50 mL.
  d. Spin down 350 × *g* for 7 min at 4°C.
  e. Remove the supernatant.
  f. Resuspend the cells in 3 mL RPMI 1640. This suspension is referred to as a “monocyte-enriched suspension.”
  g. Count the cells with a hemocytometer, or an automated cell counter system such as a CASY cell counter or a Sysmex hematology instrument.
  h. ***Optional:*** Determine cell purity by Flow Cytometry (CD45^+^ CD14^+^ cells). Monocyte enrichment usually reaches 40%–80%.

3. Separation of monocytes from lymphocytes
  a. Bring isolated PBMCs to a concentration of 50–70 × 10^6^ cells per mL of RPMI 1640.
  b. Add 10 mL of hyper-osmotic Percoll solution to a 15 mL conical tube.
  c. Slowly add 3 mL of the PBMC suspension on top of the Percoll solution with a 1 mL pipet: Slightly tilt the tube and put the pipet point on the wall of the tube (approximately 1 cm above the Percoll solution). Slowly release the fluid. Be careful with the first drops, afterwards you can slowly increase the speed (Methods video S2).
  d. Spin down 580 × *g* for 15 min at 20°C acceleration 1, no break.

4. Isolation of monocyte fraction
  a. Take the interphase layer using a sterile Pasteur pipette (Figure 2).

Methods video S2. How to lay the PBMC suspension over the Percoll layer, related to monocyte isolation with Percoll

## Key resources table

**Table:** 

REAGENT or RESOURCE	SOURCE	IDENTIFIER
**Antibodies**		

FITC-conjugated CD14	Beckman Coulter	Item# B36297
APC-conjugated CD45	Beckman Coulter	Item# IM2473

**Biological samples**		

Buffy coat	this study	n/a
Human pooled serum	this study	n/a

**Chemicals, peptides, and recombinant proteins**		

Phosphate buffered saline (1×) without calcium and magnesium	Lonza	CAT# BE17-516F
RPMI 1640 medium (Dutch modified)	Life Technologies	CAT# 22409031
Gentamycin	Thermo Fisher	CAT# 15750060
Percoll	Sigma-Aldrich	CAT# P1644
Ficoll-Paque	GE Healthcare	CAT# 17-1440-03
β1,3-(D)glucan (β-glucan)	Saeed et al., 2014	N/A
Sodium pyruvate	Life Technologies	CAT# 11360088
Glutamax supplement	Life Technologies	CAT# 35050087
Sodium chloride	Sigma-Aldrich	CAT# S5886
Trypan blue solution	Sigma-Aldrich	CAT# T8154

**Critical commercial assays**		

CytoTox 96 Non-Radioactive Cytotoxicity Assay	Promega	CAT# G1780

## Step-by-step method details

### *In vitro* induction of trained immunity in adherent monocytes

**Timing: 7 days**

Before starting: heat up the PBS at 37°C (warm PBS). Complete RPMI 1640 medium with Glutamax (final concentration 2 mM), pyruvate (final concentration 1 mM) and gentamicin (final concentration 50 μg/mL) and heat it up at 37°C (warm RPMI). RPMI complete medium is referred as “RPMI+”.

1. Day 0
  a. Prepare a suspension of 10^6^ cells per mL in RPMI+. Add 100 μL of cell suspension per well in a sterile 96-well clear flat bottom polystyrene tissue culture-treated plate. When testing a new stimulus: Add at least two technical replicates per stimulus to normalize the number of cells and the cytokine values at the end of the protocol.
  b. Incubate the plate for 1 h at 37°C 5% CO_2_ in a tissue culture CO_2_ incubator to let the monocytes adhere.
  c. After 1 h, remove the supernatant that contains the non-adherent cells and discard it.
  d. Wash off remaining non-adherent cells: Add 200 μL warm PBS per well and then discard it. After this step the purity of monocytes on the plate is >90% (see Bekkering et al., 2016).
  e. Add 200 μL of the corresponding trained immunity-inducing stimuli in warm RPMI+ supplemented with 10% human pooled serum per well. Take RPMI+ as a negative control and 1 μg/mL β-glucan or 5 μg/mL BCG as a positive control.
  f. Incubate 24 h at 37°C, 5% CO_2_.

***Note:*** The different components of the serum can interact with the stimuli employed (e.g., opsonization) affecting the survival of the cells and/or their ability to induce trained immunity. Therefore, we recommend to try this protocol with different concentrations of serum (0%–10%) every time a new stimulus is tested and compare the percentages of apoptosis/cell death in the wells treated with different concentrations of serum over the length of the experiment (at each washing or change of medium step: day 1, day 3, and day 6).

***Note:*** PBS should contain calcium. The use of PBS without calcium (or with EDTA) can initiate the detachment of the cells from the well.

***Note:*** Human serum in this procedure is not heat-inactivated, even though other authors have successfully employed heat-inactivated serum (Leonhardt et al., 2018).

2. Day 1
  a. Discard supernatant or use for cytokine measurement and/or LDH cytotoxicity assay.
  b. Wash off remaining stimuli with 200 μL of warm PBS and add 200 μL warm RPMI+ supplemented with 10% pooled human serum.
  c. Incubate for 6 days, refresh medium once at day 3.

***Note:*** Use PBS without EDTA for the washing step to prevent detaching of the cells.

***Note:*** It is expected that some cells might detach or undergo apoptosis during the differentiation process.

3. Day 3
  a. Discard supernatant or use for cytokine measurement and/or LDH cytotoxicity assay.
  b. Add 200 μL warm RPMI+ supplemented with 10% pooled human serum.
4. Day 6
  a. Discard supernatant or use for cytokine measurement and/or LDH cytotoxicity assay. Add 200 μL warm RPMI+ with a secondary stimulus, for example RPMI+ (negative control), 10 ng/mL of lipopolysaccharide (LPS) from *E. coli* or 10 μg/mL of Pam3Cys.
  b. Incubate for 24 h at 37°C, 5% CO_2_.

***Note:*** Day 6 supernatants can also be stored for the determination of lactate.

5. Day 7
  a. Centrifuge the plates 350 × *g* 8 min to spin down cells that detached during the incubation period. Collect 180 μL of supernatants and use immediately or freeze at −20°C for assessment within 2 to 4 weeks, or at −80°C for further assessment. The responsiveness of the cells to heterologous secondary stimulation can be easily assessed by means of ELISA (TNFα or IL-6 levels in the supernatant) (troubleshooting 3).

***Note:*** Different incubation times and starting cell amounts might be considered depending on the final read-out (FACS, qPCR, ELISA). When new stimuli are tested, there should be an extra step at day 7 to evaluate the live cell numbers per well to normalize the cytokine results to the cell numbers:

#### Counting live cells with trypan blue

Cells can be detached from the well by incubating them with cold PBS with 2 mM EDTA for 10 min at 4°C. After this, the cells can be collected from the well by pipetting. Dilute the cells 1:5 with Trypan Blue and count the cells non-stained in blue in a hemocytometer.

#### Assessing cytotoxicity

If the numbers of cells are different, the cytokine production for that stimulus should be normalized per cell number. LDH cytotoxicity assay needs to be performed with fresh supernatants (not frozen) following the instructions of the manufacturer. The LDH cytotoxicity assay should include per cell type and concentration a positive control of cell lysis and a negative control (medium control), in order to calculate the percentage of cytotoxicity. A difference lower than 10% cytotoxicity between the conditions can be considered as acceptable.

6. Day 7 or later: Assessment of cytokine levels by ELISA.
  Perform ELISA following the instructions of the manufacturer. The concentration of cytokines in the supernatant is usually above the detection limit of the commercial kits, so the samples need to be diluted in the dilution buffer specified by the manufacturer. For R&D Duoset ELISA kits, the dilution range of the supernatants from monocytes trained with β-glucan or BCG and restimulated with LPS at day 6 is 10×–20× dilution for TNFα and 25×–100× dilution for the detection of IL-6. The dilutions need to be optimized per stimulus and might differ between laboratories, batches of stimuli, and donors. The appropriate controls for the assay are the following:
  - Non-trained, non-rechallenged cells: No detectable cytokine production
  - Trained, non-rechallenged cells: No detectable cytokine production
  - Non-trained, rechallenged cells: High cytokine production
  - Trained, rechallenged cells: Very high cytokine production

**Figure 3:**
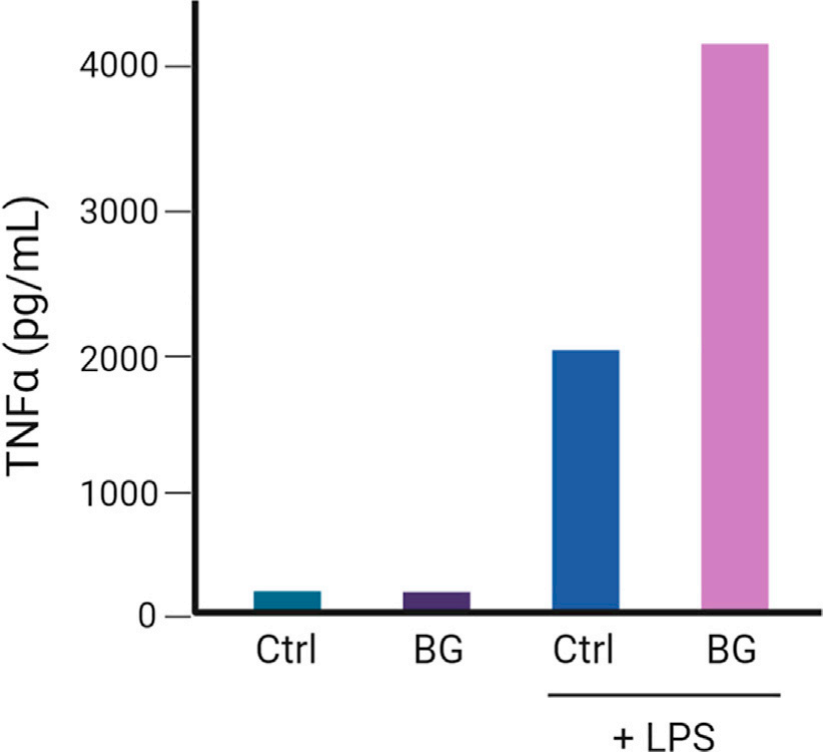
Expected cytokine production by human monocytes after the induction of trained immunity *in vitro* and secondary restimulation with LPS Ctrl, non-trained cells; BG, cells stimulated with β-glucan at day 0; LPS, cells rechallenged with LPS at day 6.

The magnitude of the induction of trained immunity will correspond to the increase of the production of TNFα or IL-6 between the non-trained, rechallenged cells and trained-rechallenged cells (Figure 3).

***Note:*** Other methods such as cytometric bead array or bioplex with a wide detecting range of multiple cytokines may also be used to avoid dilution of the samples.

## Expected outcomes

After the induction of trained immunity in monocytes, the cells that have been exposed briefly to the first challenge with β-glucan or BCG will present increased production of IL-6 and TNFα after LPS or Pam3Cys restimulation than cells that were not exposed to a trained immunity-inducing stimulus (Figure 3).

## Quantification and statistical analysis

This protocol allows the use of paired samples. Cells from the same donor will be challenged with different stimuli and the production of cytokines by the cells of the same donor can be compared. Cytokine production generally is non-parametric. In this regard, you can perform a non-parametric, paired analysis to compare the values of cytokine production after restimulation of the cells. Therefore, you can employ the Wilcoxon signed-rank test to test the hypothesis that the cytokine production of trained cells after restimulation is different than that of non-trained cells.

## Limitations

This is an *in vitro* approach for the induction of trained immunity with different stimuli. It is an alternative to *in vivo* approaches but does not substitute it.

Natural cell death may occur during the experiment depending on the stimulus employed, this is why the evaluation of cell death together with the estimation of cell number in the wells ensures a correct estimation of the trained immunity effect.

This protocol is optimized for its use in human primary monocytes. Its application to other cell types susceptible of undergoing trained immunity, such as natural killer cells, dendritic cells, or mouse cells, would need further adjustments and optimization. The protocol also needs to be amended when used in (human) cell lines such as THP1 monocytes.

In our protocol we employ human pooled serum obtained in-house. Other laboratories successfully employ similar protocols using commercial sera from different sources, such as 10% (heat-inactivated) human AB serum (Sigma-Aldrich, H3667) (Leonhardt et al., 2018). The use of a different source of serum (including FCS) might require further adjustment of the concentrations employed to achieve an optimal induction of trained immunity.

We employ β-glucan kindly provided by Prof. David Williams (East Tennessee State University). Other laboratories have successfully employed β-glucan from different sources such as Beta-glucan peptide (Invivogen, Cat#tlrl-bgp) in mice (Kalafati et al., 2020). However, the suitability and trained immunity-inducing capacities of the alternative sources of β-glucan needs to be tested per laboratory and experimental setup.

## Troubleshooting

### Problem 1

Inadequate separation of layers during Ficoll/Percoll isolation (related to Before your begin session)

### Solution 1

Make sure that the acceleration and break settings of the centrifuge are set correctly. At high speed, the phases might mix and disturb the gradient. Remember to slowly lay the monocyte suspension on the Percoll/Ficoll solution. Make sure that your concentration of monocytes or the amount of blood per tube does not exceed the recommendations. The Ficoll-Paque PLUS reagent and the Percoll solution should be at room temperature.

### Problem 2

Too much clumping of the cells during the separation:

### Solution 2

You can consider to add 2 mM sterile EDTA to your PBS. This will reduce the clumping. Make sure that the addition of EDTA does not interfere with the subsequent assays.

### Problem 3

Lack of induction of trained immunity:

### Solution 3

The most likely is a contamination of the trained immunity-inducing stimulus with e.g., LPS. If the stimuli employed for the first challenge are contaminated, this can cause the cells to become unresponsive to any secondary stimulation. If you are using BCG to induce trained immunity, make sure it is freshly reconstituted, it can lose its ability to induce trained immunity within a few days after reconstitution. Recent vaccinations or infections can also induce alterations in the induction of trained immunity in monocytes. Please also note that it is known that there is profound interindividual variation in training capacity, which depends, at least in part, on genetic variation in proteins involved in the mechanisms that drive trained immunity.

### Problem 4

Variability between batches of stimuli:

### Solution 4

Diverse batches of stimuli present different abilities to induce trained immunity. This can affect both the challenge employed for the first challenge and the second. Every time we employ a new batch of any given stimulus we should compare cell survival, the ability to induce trained immunity and the cytokine production of the cells compared to the previous batch and adjust accordingly the concentrations.

### Problem 5

Variability depending on the time of the year (seasonality of the results):

### Solution 5

It is known that the behavior of the immune responses varies depending on the time of the year, affecting cytokine responses (Ter Horst et al., 2016). In general, cytokine responses decrease during winter months, which is probably due to the increased incidence transmission of influenza and other diseases, which alter the cytokine production patterns. In this regard, it is recommended to keep a register of the different cytokine responses observed during the year to estimate the impact of seasonality in your experiments.

## Resource availability

### Lead contact

Further information and requests for resources and reagents should be directed to and will be fulfilled by the lead contact, Mihai Netea (mihai.netea@radboudumc.nl).

### Materials availability

This protocol does not generate new unique reagents.

### Data and code availability

This protocol does not generate or analyze any datasets or codes.
